# Research trends and frontiers of astrocytes in cognitive impairment: a bibliometric analysis from 2015 to 2024

**DOI:** 10.3389/fnagi.2025.1708008

**Published:** 2026-01-13

**Authors:** Zhilin Huang, Wanting Liu, Yating Zhang, BiXiang Zha, Ping Wang, Jun Yang

**Affiliations:** 1 The First Clinical Medical College of Anhui University of Chinese Medicine, Hefei, China; 2 The First Affiliated Hospital of Anhui University of Chinese Medicine, Hefei, China

**Keywords:** astrocytes, bibliometric, CiteSpace, cognitive impairment, multi-database validation, VOSviewer

## Abstract

**Objective:**

Astrocytes, constituting the predominant glial cell population with in the central nervous system, have emerged as a focal point of investigation due to their multifaceted roles and therapeutic implications in cognitive disorders. Despite the growing body of research, there has yet to be a bibliometric analysis to determine research trends and hotspots in this field.

**Methods:**

We searched for publications related to cognitive impairment and astrocytes in the Web of Science Core Collection (WoSCC), PubMed, and Scopus databases from January 1, 2015, to December 31, 2024. Using VOSviewer, CiteSpace software, and bibliometrix based on the R programming language, we performed visualization and bibliometric analysis of WoSCC data, covering aspects such as countries, institutions, authors, journals, keywords, and references. Additionally, we conducted equivalent searches in the Scopus and PubMed databases using the same keyword combinations, time range, and screening criteria. By verifying the consistency of time series, thematic focus, and country rankings across databases, we ensured the stability and universality of the results.

**Results:**

Over the past decade, investigations into the role of astrocytes in cognitive disorders have demonstrated a consistent upward trajectory, with the United States and China emerging as leading contributors. The primary focus has been on Alzheimer’s disease, Parkinson’s disease, VD, and Traumatic brain injury. The hippocampus has been identified as a critical brain region in these studies. Neuroinflammation has persisted as a central research focus and continues to represent a key direction for future investigations. Synaptic dysfunction is a recent research hotspot. The integration of single-cell sequencing technology has facilitated more comprehensive mechanistic analyses in this field. Multi-database validation results indicate that publication trends, thematic priorities, geographical distribution, and journal distribution exhibit macro-level stability.

**Conclusion:**

This study employed bibliometric methods to map the development trends and research hotspots in studies related to astrocytes and cognitive impairments over the past decade, and emphasized the importance of translating current research into clinical applications. This will provide insights and references for future studies.

## 1 Introduction

Cognitive impairment is a common clinical syndrome characterized by a significant decline in one or more core cognitive domains, including memory, executive function, attention, language, visuospatial abilities, and social cognition (First, 2013). This condition spans a spectrum from mild cognitive impairment to severe dementia, constituting a fundamental feature of various neurodegenerative disorders, including Alzheimer’s Disease (AD), Vascular Dementia (VD), Dementia With Lewy Bodies (DLB), Frontotemporal Dementia (FTD), as well as certain neurodevelopmental conditions (Alam et al., 2018; Morrow et al., 2023). Cognitive impairment significantly compromises patients’ functional abilities, imposing substantial personal, familial, and societal burdens. Moreover, with the progression of global population aging, its prevalence exhibits a marked upward trajectory (Livingston et al., 2020). These factors underscore the critical need for identifying and validating therapeutic targets to address this multifaceted condition.

Astrocytes (AS) constitute the most abundant and widely distributed glial cell population within the central nervous system (Allen and Lyons, 2018). Once primarily regarded as passive supportive elements, these cells have now emerged as critical contributors to the pathophysiological mechanisms underlying cognitive disorders (Santello et al., 2019). This marks a significant shift in researchers’ understanding of astrocytes. Astrocytes assist neurons with their structure and metabolism, and they also play a significant role in important physiological and pathological processes, such as synapse formation, neurotransmitter cycling (e.g., glutamate homeostasis), ion balance, blood–brain barrier (BBB) regulation, immune surveillance, and neuroinflammatory responses (Khakh and McCarthy, 2015; Sofroniew and Vinters, 2010). Recent evidence indicates that these pathological processes mediated by astrocytes participate in the progression of cognitive impairment and exhibit significant heterogeneity and plasticity (Escartin et al., 2021; Pekny et al., 2016).

However, there is currently no research that employs bibliometric methods, utilizing statistical and computational techniques to reveal the macro development patterns, research hotspots, collaborative networks, and emerging frontiers in this field, thereby providing insights into the development trends of complex research areas. Therefore, this study aims to provide a clear “research map” for researchers and clinicians in the field by conducting a systematic quantitative analysis of the literature on astrocytes in the field of cognitive impairment over the past decade, thereby offering valuable decision-making references for subsequent research.

## 2 Method

### 2.1 Data source and search strategy

**Figure 1:**
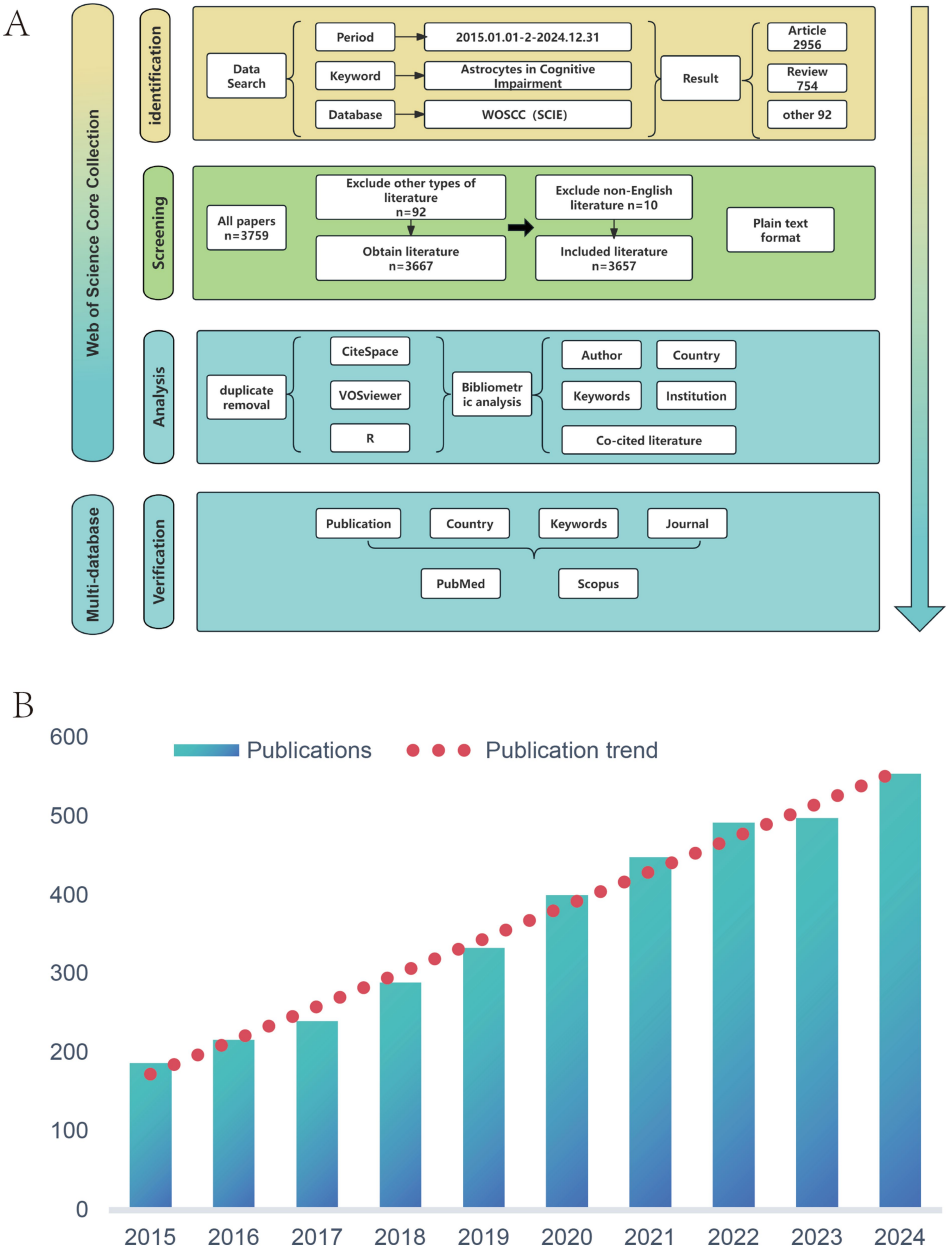
**(A)** Flowchart of data retrieval and analysis. **(B)** The annual publication volume and development trend of publications from 2015 to 2024.

The literature search was conducted using the Web of Science Core Collection (WoSCC), which has stringent inclusion criteria. Collecting literature from this database helps avoid the inclusion of studies with low research value, thereby ensuring the accuracy of this study (Liu et al., 2025; Veiga-Del-Bano et al., 2023; Zhang et al., 2021). In this research, the Science Citation Index Expanded (SCIE) was selected. After discussion, the following search strategy was determined: (TS = (Astrocytes OR Astrocyte OR “Astroglia Cells” OR “Astroglia Cell” OR “Cell, Astroglia” OR “Astroglial Cells” OR “Astroglial Cell” OR “Cell, Astroglial” OR Astroglia OR Astroglias OR Astroglial)) AND (TS = ((“Cognitive Dysfunction”) OR (“Cognitive Dysfunctions”) OR (“Dysfunction, Cognitive”) OR (“Dysfunctions, Cognitive”) OR (“Cognitive Disorder”) OR (“Cognitive Disorders”) OR (“Disorder, Cognitive”) OR (“Disorders, Cognitive”) OR (“Cognitive Impairments”) OR (“Cognitive Impairment”) OR (“Impairment, Cognitive”) OR (“Impairments, Cognitive”) OR (“Mild Cognitive Impairment”) OR (“Cognitive Impairment, Mild”) OR (“Cognitive Impairments, Mild”) OR (“Impairment, Mild Cognitive”) OR (“Impairments, Mild Cognitive”) OR (“Mild Cognitive Impairments”) OR (“Cognitive Decline”) OR (“Cognitive Declines”) OR (“Decline, Cognitive”) OR (“Declines, Cognitive”) OR “cognitive deficit” OR “cognitive deficits” OR dementia OR dementias)). Over the past decade, advancements in single-cell sequencing technology and reduced costs have significantly facilitated research into the functional heterogeneity of astrocytes, driving vigorous development in this field (Patani et al., 2023). Our preliminary analysis confirms this trend: before 2015, research in this area remained relatively fragmented, with annual publications typically falling below 200. However, since 2015, the number of papers has shown a marked upward trend, surging from 187 in 2015 to 554 in 2024. Through group discussions, we determined the search period for bibliometric analysis to be from January 1, 2015, to December 31, 2024. This timeframe not only covers the current peak period of research activity and densest output but also effectively captures key nodes and frontier trends in the evolution of the discipline. Data was exported in plain text format, with the detailed filtering process illustrated in Figure 1A.

### 2.2 Bibliometric analysis

For comprehensive bibliometric analysis and visualization, we utilized three established analytical tools: VOSviewer (version 1.6.20), CiteSpace (version 6.4. R1), and bibliometrix (Aria and Cuccurullo, 2017; Chen, 2004; van Eck and Waltman, 2010). The analysis was conducted from multiple dimensions, including countries, institutions, journals, authors, keywords, and references, to elucidate the current research landscape and emerging trends within this scientific domain.

### 2.3 Multi-database validation

To verify the robustness and comprehensiveness of the results, we employed an equivalent search strategy in the PubMed and Scopus databases, including the same keywords, Boolean operators, and time frame (from January 1, 2015, to December 31, 2024). The specific search strategies were adapted according to the retrieval logic of each database (see Supplementary Table 1 for details). Inclusion and exclusion criteria were kept consistent with those of the WoSCC to ensure the comparability of the results. We extracted several key metrics from the bibliometric analysis, including the total number of retrieved records, annual publication trends, major contributing countries, high-frequency author keywords, and core journals. We compared these metrics with the original WoSCC dataset. We employed Pearson’s correlation coefficient to analyze the consistency of annual publication trends across different databases. Furthermore, we utilized the Jaccard similarity coefficient to evaluate the consistency in the composition of keywords, primary contributing countries, and core journals between different databases. Spearman’s correlation analysis was commonly applied to assess the consistency in the relative rankings of keywords and countries. This cross-database comparison validated whether the thematic focus, geographical distribution, and temporal patterns observed in the WoSCC analysis could be reproduced in other major literature sources.

## 3 Result

### 3.1 Annual publication volume and trend analysis

From 2015 to 2024, the annual number of publications exhibited a sustained and significant upward trend, demonstrating overall stable development. By 2024, the number of publications had risen to approximately 550, reaching a 10-year high. The red trend line (Publication trend) clearly reflects this long-term growth trajectory, indicating that academic output in this field is not only expanding continuously but also exhibits strong regularity and sustainability (Figure 1B). This may be attributable to increased research investment, the expansion of scholarly teams, and the innovative impetus generated by interdisciplinary integration.

### 3.2 Country analysis

**Figure 2:**
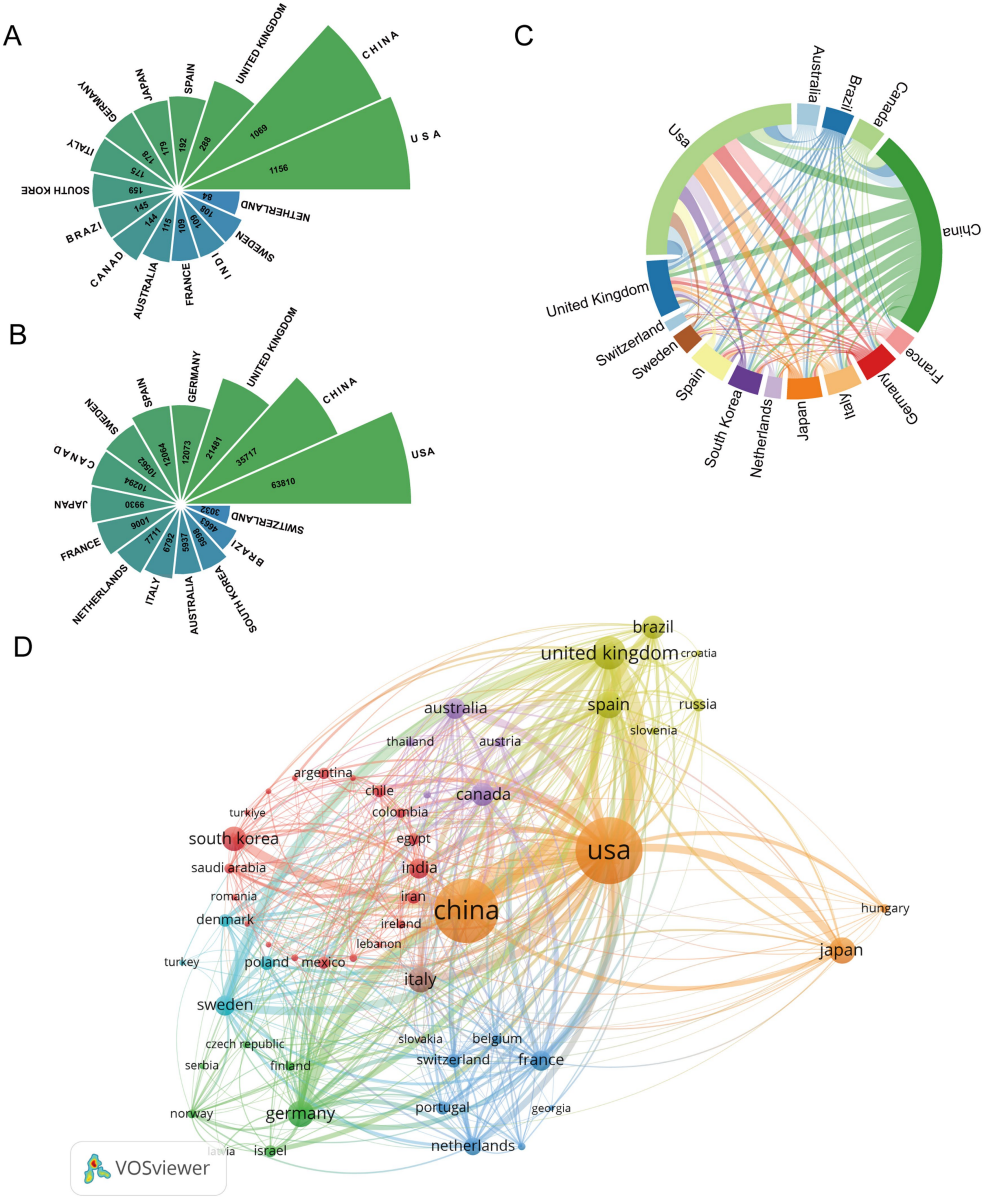
Visual analysis of the country. **(A)** The top 10 countries in terms of the number of published articles; **(B)** the top 10 countries in terms of citation count; **(C)** chord chart of the top 20 countries’ cooperation networks in terms of the number of published articles. Each arc segment represents a country. The width of the arc segment indicates that the country is directly proportional to the number of publications. The colored lines connecting two arc segments represent the intensity of scientific research cooperation between the two countries. The thicker the lines, the more frequent the cooperation between the two countries; **(D)** use VOSviewer to generate the visualization of the national cooperation network.

It is shown in the figure that during the period from 2015 to 2024, international cooperation in the field of astrocytes and cognitive disorders has exhibited a clear ‘bipolar’ characteristic: with the United States and China at the core, reaching out to numerous countries worldwide. As illustrated (Figures 2A,B), among the top 15 countries ranked by publication volume and citation frequency, the United States, acting as a global research hub, published 1,156 papers which were cited 63,810 times, establishing strong cooperative relationships with over 20 countries, including China, Germany, and Japan. Meanwhile, China plays a pivotal role in the Asia-Pacific region, publishing 1,069 papers cited 35,717 times, especially deepening cooperation with Japan, South Korea, and Australia. European countries have formed a relatively independent yet highly cohesive network of cooperation centered around Germany, France, and the Netherlands, reflecting a high degree of integration of regional scientific research resources (Figures 2C,D). Notably, despite Sweden’s publication volume (108 papers) ranking only 14th, its total citation count (10,562 citations) is among the highest, with an impressive average of 97.8 citations per paper. This significant ‘high impact, low quantity’ characteristic strongly indicates that Sweden’s research in this field has extremely high academic quality and international influence. This exceptional impact likely stems from concentrated investments in top institutions such as the Karolinska Institutet.

### 3.3 Institutional analysis

**Figure 3:**
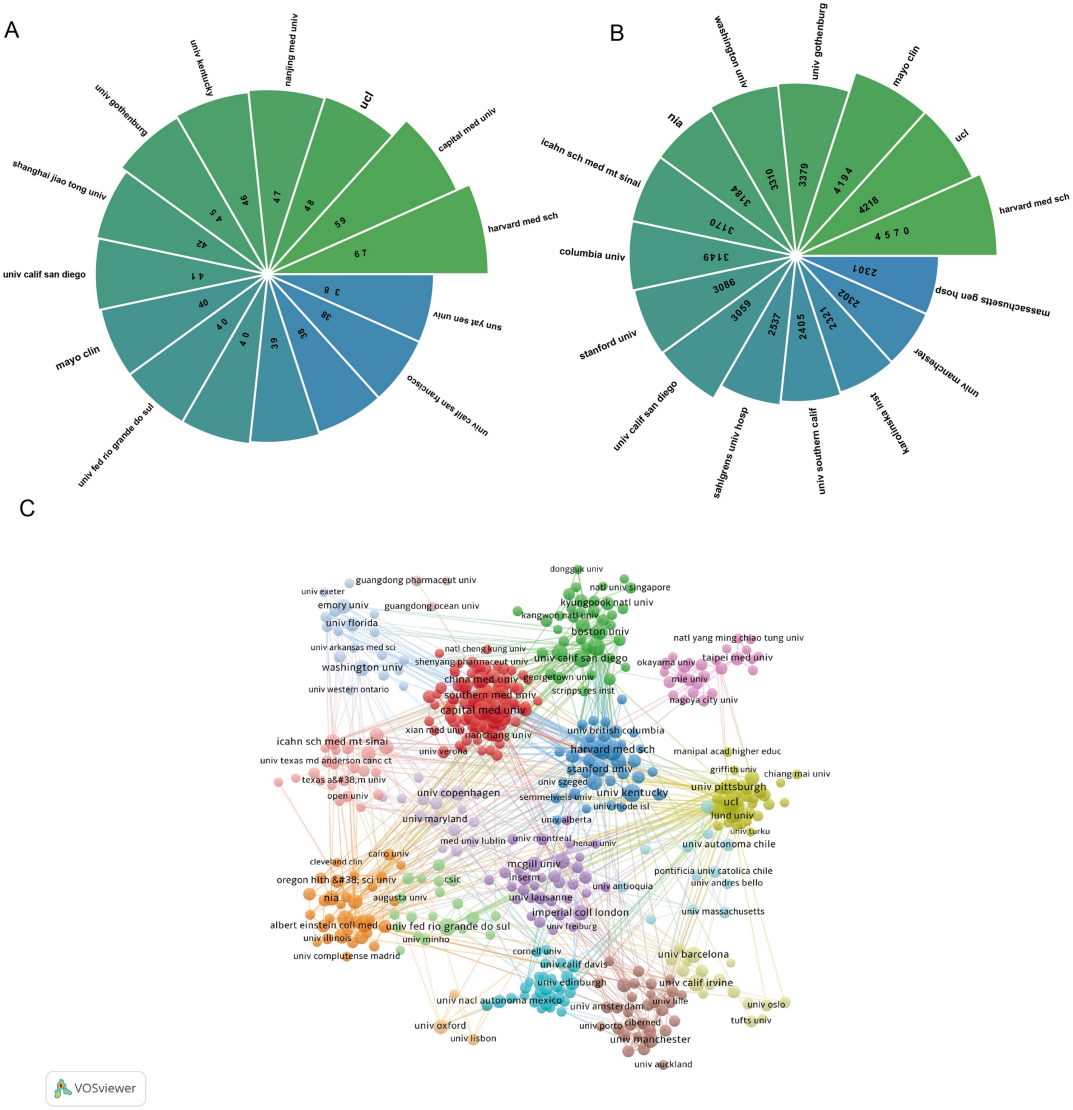
Visual analysis of institutions. **(A)** Top 10 institutions in terms of the number of published articles; **(B)** top 10 institutions in terms of citation count; **(C)** use VOSviewer to generate visual analyses of over 10 institutional collaboration networks for publications. Nodes of different colors represent institutions in different color clusters, and the size of the nodes indicates their frequency of occurrence.

The comprehensive analysis of 3,962 participating institutions shows that Harvard Medical School has the highest research output, publishing 67 papers that have been cited 4,570 times (Figures 3A,B). With top universities in Europe and America as the core of cooperation, it forms a dense international research alliance, dominating the academic discourse and research direction in this field. Notably, among the top 15 institutions ranked by publication volume, four were affiliated with China, reflecting substantial contributions from Chinese academic institutions to this research domain. However, in terms of citation impact, 9 of the top 15 institutions were based in the United States, consistent with the national influence analysis and indicative of the United States’ dominant academic leadership in this field. Furthermore, institutional collaboration network analysis demonstrated the establishment of robust cooperative relationships among participating institutions (Figure 3C).

### 3.4 Author analysis

**Figure 4:**
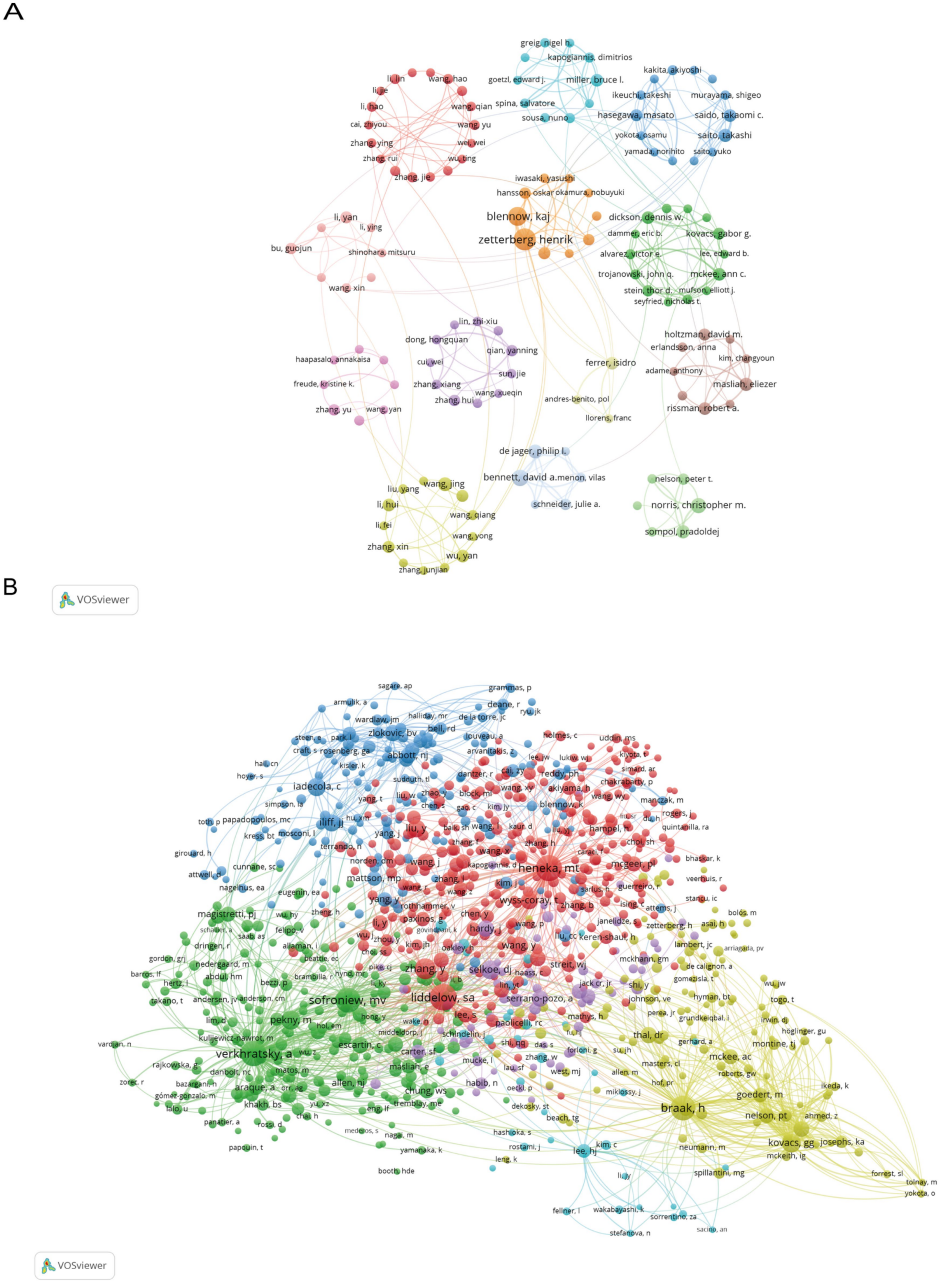
The author’s visual analysis. **(A)** Use VOSviewer to generate a visual analysis of the author collaboration network of over 10 publications. **(B)** Visual analysis of co-cited authors with more than 10 citations.

Table 1 lists the top 10 authors in terms of publication volume and co-citation count. Henrik Zetterberg is a leading scholar in this field, with the highest number of publications (28). From the collaboration network diagram, it can be observed that the field has formed multiple collaborative groups centered around Henrik Zetterberg, Jing Wang, Alexei Verkhratsky, and David A. Bennett (Figure 4A). Liddelow, S. A., with 652 co-citations, is the most frequently co-cited author. Figure 4B shows a visualization of co-cited authors generated using VOSviewer.

**Table 1:** The top 10 authors in terms of the number of articles published and the number of citations.

Author	Publications	Author	Citations
Zetterberg, Henrik	28	Liddelow, Sa	652
Blennow, Kaj	22	Heneka, Mt	618
Felipo, Vicente	20	Verkhratsky, A	578
Goncalves, Carlos-Alberto	17	Sofroniew, Mv	546
Bennett, David A.	16	Braak, H	540
Llansola, Marta	16	Zhang, Y	374
Verkhratsky, Alexei	16	Kovacs, Gg	299
Norris, Christopher M.	14	Selkoe, Dj	290
De Jager, Philip L.	12	Pekny, M	282
Wang, Jing	12	Iadecola, C	268

### 3.5 Journal analysis

**Figure 5:**
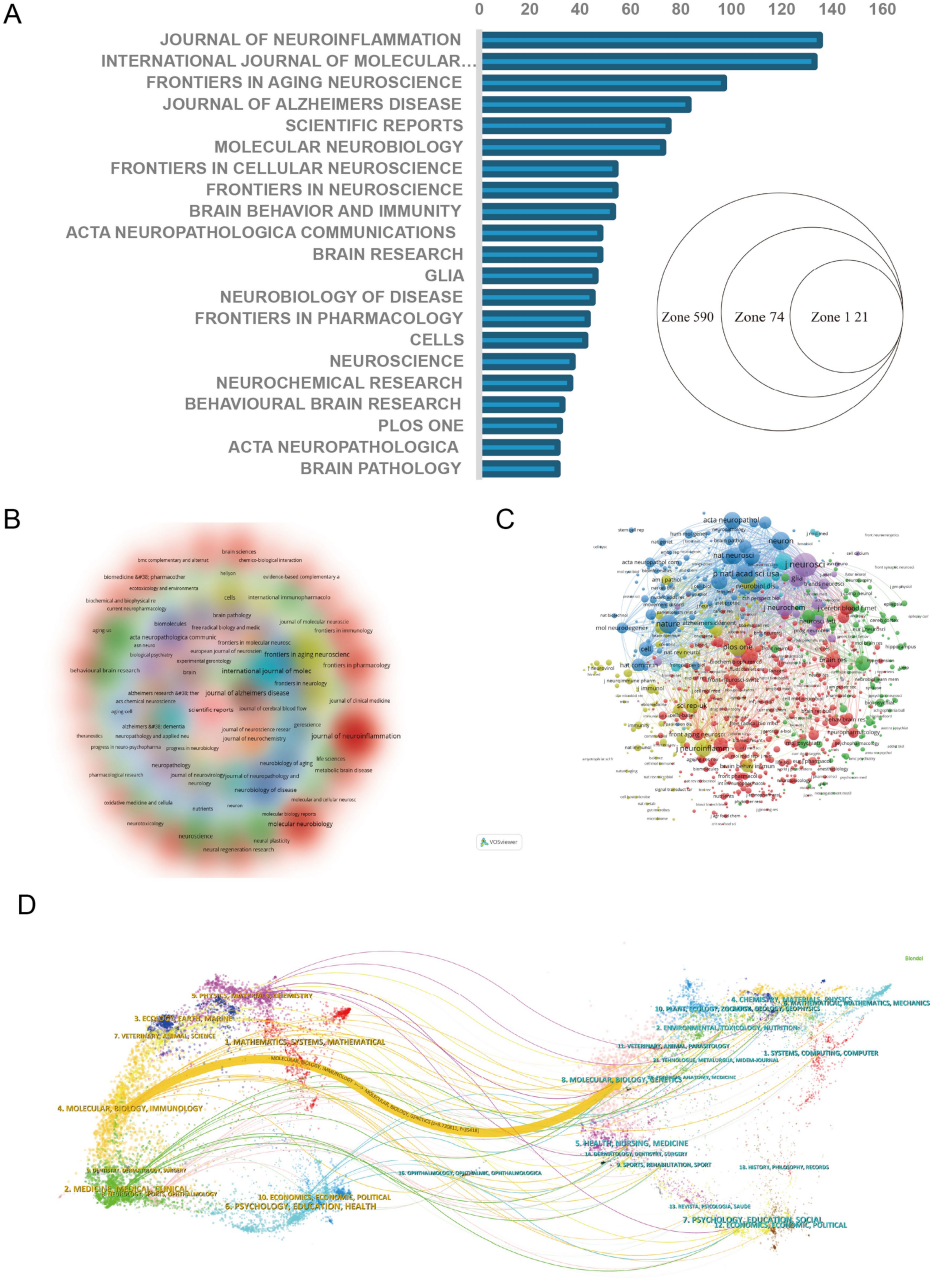
Visual analysis of journals. **(A)** Publication volume of core journals plotted according to Brad’s law. Zones 1, 2, and 3 represent core journals, related journals, and non-related journals, respectively. **(B)** Co-occurrence maps for journals; **(C)** co-occurrence maps for co-cited journals; **(D)** journal double-stacked plot.

Empirical studies in scientometrics have demonstrated that seminal papers within a discipline are predominantly published in the field’s core journals. Utilizing Bradford’s Law (Fernandez-Llimos, 2016), we identified 21 core journals in this field. Notably, the Journal of Neuroinflammation has become the journal with the highest number of publications, totaling 135 articles (Figures 5A–C). These 21 core journals cover multiple disciplines such as neuroscience, molecular biology, neuroimmunology, geriatrics, and cellular pathology, reflecting the multidimensional roles and interdisciplinary nature of astrocytes in cognitive impairment research. It is noteworthy that the inclusion of high-impact journals such as Brain Behavior and Immunity and Acta Neuropathologica Communications indicates significant breakthroughs in the field of astrocyte-related cognitive disorders. The publication of these research findings not only enhances the importance of this field but also lays a solid foundation for future scientific research and clinical applications. Tables 2, 3 list the top 10 journals ranked by the number of publications and citation frequency, respectively. The Journal of Neuroscience is the most cited.

**Table 2:** The top 10 journals in terms of the number of published articles.

Rank	Journal	Publications	IF(2024)
1	Journal Of Neuroinflammation	135	10.1
2	International Journal Of Molecular Sciences	133	4.9
3	Frontiers In Aging Neuroscience	97	4.5
4	Journal Of Alzheimer’s Disease	83	3.1
5	Scientific Reports	75	3.9
6	Molecular Neurobiology	73	4.3
7	Frontiers In Cellular Neuroscience	54	4.0
8	Frontiers In Neuroscience	54	3.2
9	Brain Behavior And Immunity	53	7.6
10	Acta Neuropathologica Communications	51	5.7

**Table 3:** The top 10 co-cited journals.

Rank	Journal	Citations	IF(2024)
1	Journal of Neuroscience	9,337	4.0
2	Proceedings of the National Academy of Sciences of the United States of America	6,074	9.1
3	Nature	5,175	48.5
4	Neuron	4,939	15.0
5	Plos One	4,589	2.6
6	Neurobiology of Aging	4,339	3.5
7	Journal of Alzheimer’s Disease	4,297	3.1
8	Journal Of Neuroinflammation	4,239	10.1
9	Acta Neuropathol	4,195	9.3
10	Glia	4,052	5.1

The journal double-stack graph presents the flow of knowledge in this field (Figure 5D). The left as the cited journals, the right to lead journals, the most prominent yellow quotations from “MOLECULAR BIOLOGY, IMMUNOLOGY” to “MOLECULAR BIOLOGY, Genetics” reflects the research level from the mesoscopic level such as immune molecules to genetic and other changes in the micro level.

### 3.6 Keyword analysis

Keywords are the embodiment of the central idea of an article (Huang et al., 2025) and keyword co-occurrence analysis provides valuable insights into the developmental trajectory and current research trends regarding astrocytes in cognitive impairment. As presented in Table 4 the top 10 most frequently occurring keywords were identified. AD emerged as the most prominent keyword with a frequency of 963 occurrences underscoring its significance as a primary research focus in this domain. Following the exclusion of the search terms “astrocytes” and “cognitive dysfunction” other high-frequency keywords included neuroinflammation (659 occurrences) microglia (452 occurrences) neurodegeneration (233 occurrences) and hippocampus (207 occurrences) which collectively highlight the key areas of scientific interest and investigation in this field

**Table 4:** The top 10 high-frequency keywords.

Rank	Keyword	Frequency
1	Alzheimer’s Disease	963
2	Astrocytes	929
3	Neuroinflammation	659
4	Microglia	452
5	Cognitive Dysfunction	348
6	Inflammation	239
7	Neurodegeneration	233
8	Hippocampus	207
9	Amyloid-Beta	201
10	Dementia	193

**Figure 6:**
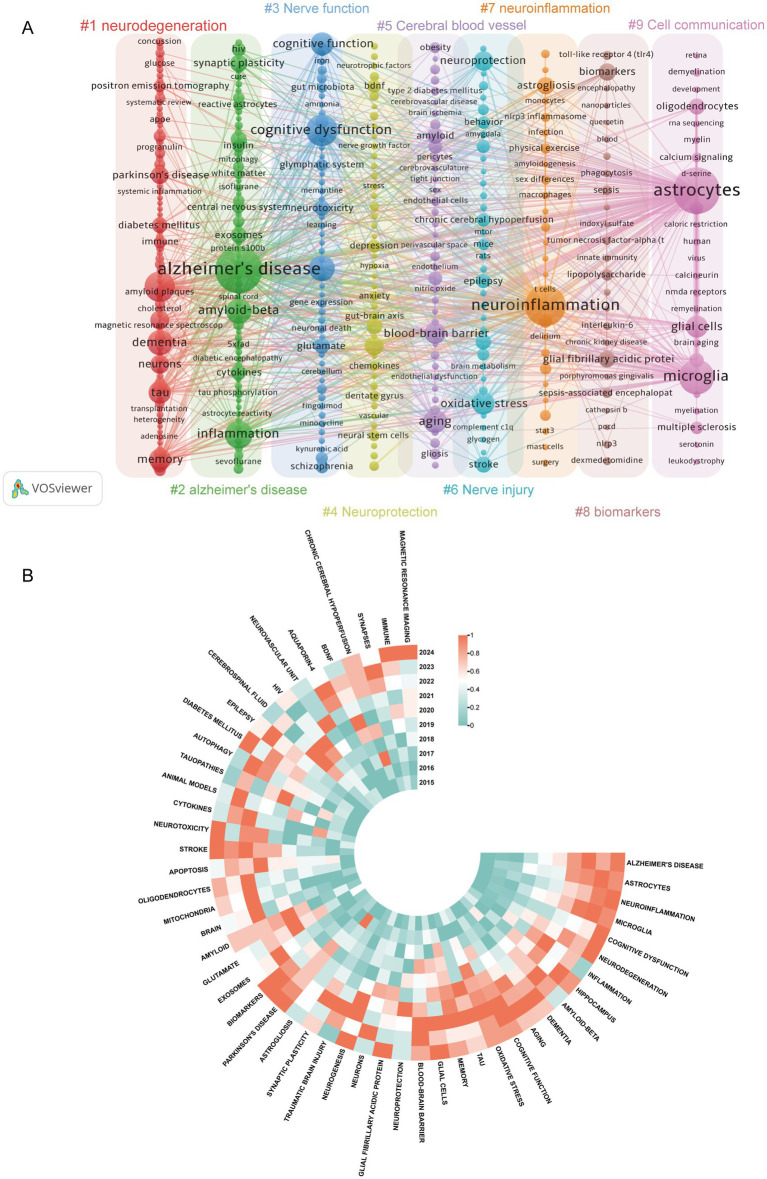
**(A)** Co-occurrence network visualization analysis of keywords in VOSviewer. Identical colors represent the same cluster. Node size indicates keyword frequency, while line thickness reflects relationship strength. **(B)** Keyword heatmap over time. Each radial line corresponds to a specific keyword, while each concentric circle represents a consecutive year. Color blocks closer to red indicate a higher frequency of that keyword in literature during that year. The closer the keywords are to Alzheimer’s disease, the higher the total frequency of their appearance.

Using VOSviewer to construct a co-occurrence network of keywords (Figure 6A) yielded nine major clusters. These clusters represent primary research directions concerning the role of astrocytes in cognitive impairment. Clusters 1 and 2 reveal several key diseases studied in this field, including PD and AD, alongside their pathological products. Cluster 3 relates to neurofunction, encompassing keywords such as glutamate energy system regulation, neurodysfunction-associated disorders, and neuroinflammatory mechanisms. Clusters 4 and 6 suggest the dual roles of astrocytes in neuroprotection and neurotoxicity. Clusters 5 and 7 indicate that cognitive impairment arising from cerebrovascular disease constitutes another key focus area, with neuroinflammation emerging as a critical pathological factor. Cluster 9 pertains to cellular communication. Figure 6B further expands the keyword analysis by introducing a temporal dimension. Over time, neuroinflammation, Alzheimer’s disease, microglia, memory, oxidative stress, and Parkinson’s disease have maintained consistently high levels of interest. Emerging focal areas over the past 3 years include neurogenesis, mitochondria, neurotoxicity, hippocampus, biomarkers, and the blood–brain barrier, reflecting shifting research priorities.

**Figure 7:**
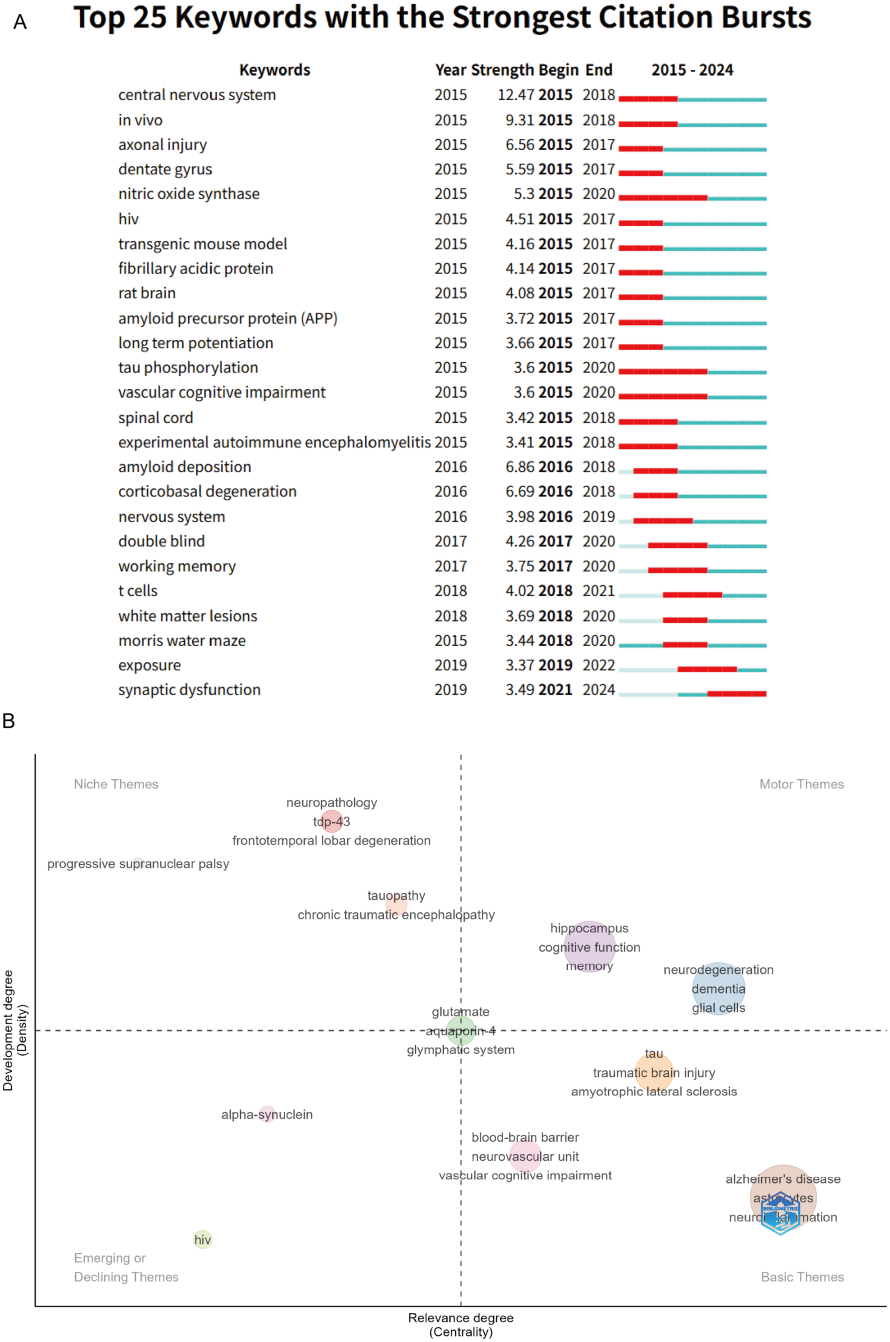
**(A)** Top 25 keywords with the strongest citation bursts; **(B)** topic evaluation chart. Related topics are divided into four quadrants. The first quadrant (upper right) represents popular and rapidly developing topics. The second quadrant (upper left) represents niche topics. The third quadrant (lower left) represents declining topics. The fourth quadrant (lower right) represents foundational topics or those yet to be fully developed.

To further analyze changes in research trends, we used Top 25 Keywords with the Strongest Citation Bursts (Figure 7A) and found that “central nervous system” was the keyword with the highest burst strength (strength = 12.47), which aligns with our focus on cognitive impairment. “Nitric oxide synthase,” “vascular cognitive impairment,” and “tau phosphorylation” have maintained long-term interest, further validating the results from Figure 6B. “Synaptic dysfunction” has shown a burst trend in the last 3 years. The thematic mapping analysis conducted using density and centrality parameters (Figure 7B) shows that vascular cognitive impairment, Alzheimer’s disease, and neuroinflammation have become the cornerstone of research in this field. Additionally, the hippocampus, neurodegeneration, and glial cells have emerged as new and hot thematic clusters within this research field.

### 3.7 Co-citation analysis

**Figure 8:**
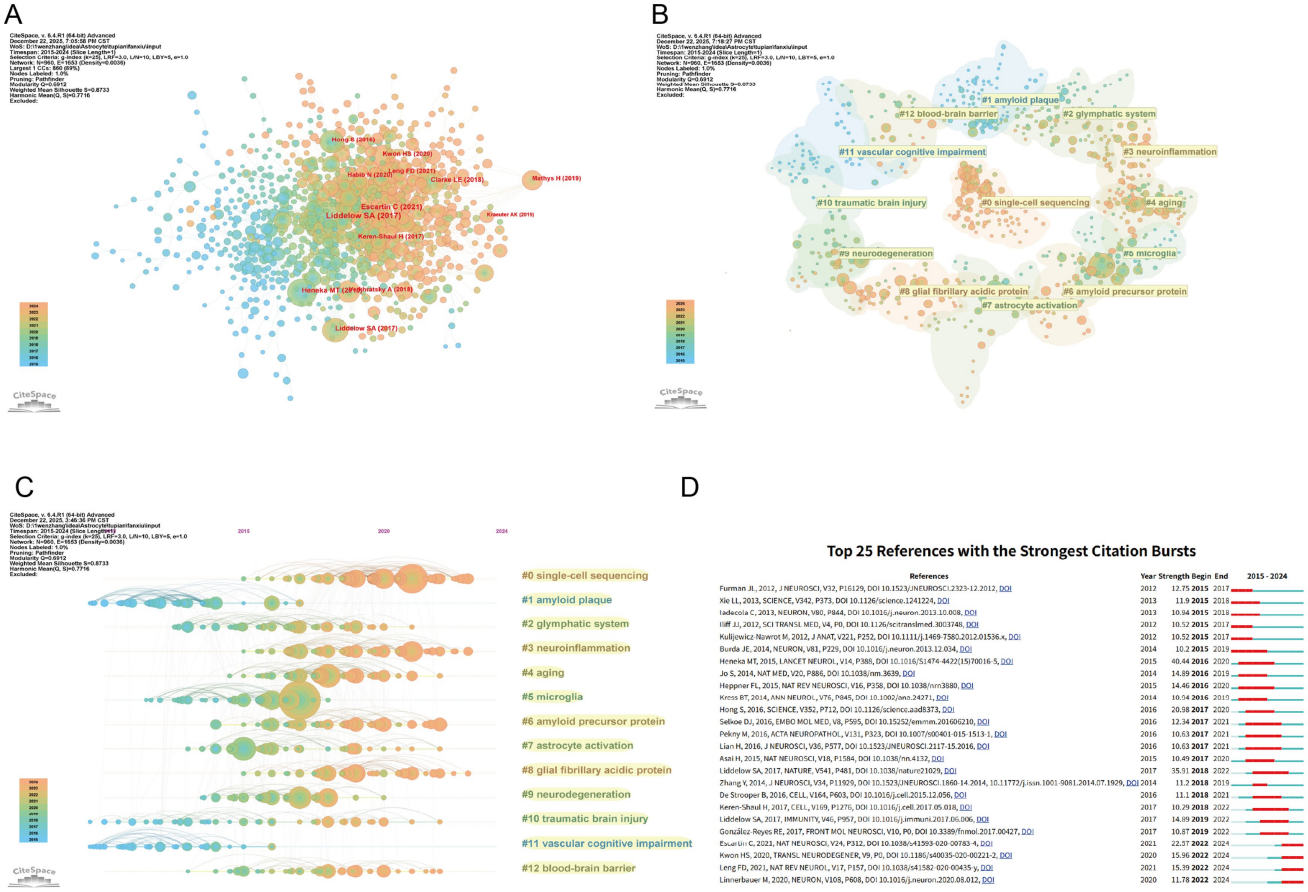
Visual analysis of co-cited literature. **(A)** Co-cited literature collaboration network generated using Citespace; **(B)** visualization of the top 13 clusters in co-cited literature analysis. Lower numbers indicate clusters containing more articles. **(C)** timeline diagram of co-cited literature clusters; **(D)** top 25 references with the highest citation burst rates. Red indicates burst duration, while burst intensity reflects attention levels during the reference period.

Analyzing co-cited literature not only helps to reveal the knowledge foundation of a discipline but also reflects the structure, evolutionary trends, and key academic contributions of the research field. Table 5 lists the top 10 articles with the highest co-citation counts. Figure 8A presents the collaboration map of co-cited literature. The most frequently co-cited article is “Neurotoxic reactive astrocytes are induced by activated microglia,” published in Nature in 2017(Liddelow et al., 2017). The study demonstrates that A1-type astrocytes exhibit neurotoxicity in various neurodegenerative diseases, while microglia can induce the formation of A1-type astrocytes.

**Table 5:** The top 10 publications in terms of total citations.

Rank	Publication	Cited	Centrality	IF
1	Liddelow SA, 2017, NATURE, V541, P481, DOI 10.1038/nature21029	292	0.09	48.5
2	Escartin C, 2021, NAT NEUROSCI, V24, P312, DOI 10.1038/s41593-020-00783-4	159	0.03	20.0
3	Heneka MT, 2015, LANCET NEUROL, V14, P388, DOI 10.1016/S1474-4422(15)70016-5	119	0.04	45.5
4	Clarke LE, 2018, P NATL ACAD SCI USA, V115, PE1896, DOI 10.1073/pnas 1,800,165,115	108	0.04	9.1
5	Liddelow SA, 2017, IMMUNITY, V46, P957, DOI 10.1016/j.immuni.2017.06.006	97	0.04	26.3
6	Leng FD, 2021, NAT REV NEUROL, V17, P157, DOI 10.1038/s41582-020-00435-y	88	0.02	33.1
7	Hong S, 2016, SCIENCE, V352, P712, DOI 10.1126/science.aad8373	84	0.03	45.8
8	Habib N, 2020, NAT NEUROSCI, V23, P701, DOI 10.1038/s41593-020-0624-8	79	0.04	20.0
9	Kwon HS, 2020, TRANSL NEURODEGENER, V9, P0, DOI 10.1186/s40035-020-00221-2	79	0.01	15.2
10	Verkhratsky A, 2018, PHYSIOL REV, V98, P239, DOI 10.1152/physrev.00042.2016	75	0.09	28.7

Through CiteSpace analysis, the co-citation network was categorized into 13 distinct clusters (Figure 8B). Cluster #1: amyloid plaque and Cluster #6: amyloid precursor protein indicate that Alzheimer’s disease is a major focus in this field, which aligns with our keyword analysis results. This highlights the importance of amyloid plaques and APP in Alzheimer’s disease research. Cluster #2: glymphatic system, Cluster #3: neuroinflammation, Cluster #4: aging, Cluster #5: microglia, and Cluster #12: blood–brain barrier reveal multiple factors contributing to cognitive impairment caused by astrocytes. These clusters illustrate various aspects of cognitive impairment mechanisms, including the function of the glymphatic system, neuroinflammatory responses, changes during aging, the role of microglia, and the integrity of the blood–brain barrier. Cluster #9: neurodegeneration, Cluster #10: traumatic brain injury, and Cluster #11: vascular cognitive impairment represent other key diseases of focus in this field. These clusters cover a broad range of neurological disorders, reflecting researchers’ interest in different types of cognitive impairments and their underlying mechanisms. The timeline analysis of co-cited literature (Figure 8C) demonstrated that cluster #0:single-cell transcriptomics has emerged as a pivotal methodological approach for elucidating the functional roles and molecular mechanisms of astrocytes in cognitive impairment.

Figure 8D displays the top 25 references with the strongest citation bursts. The study “Neuroinflammation in Alzheimer’s disease “exhibits the highest burst strength (Intensity = 40.44; Heneka et al., 2015), which reviews neuroinflammation as a crucial pathogenic mechanism and therapeutic target in Alzheimer’s disease, where the binding of pattern recognition receptors on microglia and astrocytes can trigger neuroinflammation. The consensus statement of Escartin et al. (2021) is the highly cited literature in recent years (citation intensity = 22.57), which summarizes the heterogeneity of previous studies in the definition, nomenclature, morphology, function, and other aspects of reactive AS, laying a solid foundation for subsequent research. Among the highly cited literature in the past 3 years, three articles (Kwon and Koh, 2020; Leng and Edison, 2021; Linnerbauer et al., 2020) are all related to neuroinflammation, indicating that astrocyte-mediated neuroinflammation has become a key and popular research direction in the field of cognitive impairment, and significant breakthroughs have been achieved (Table 4).

### 3.8 Results of multi-database validation

**Figure 9:**
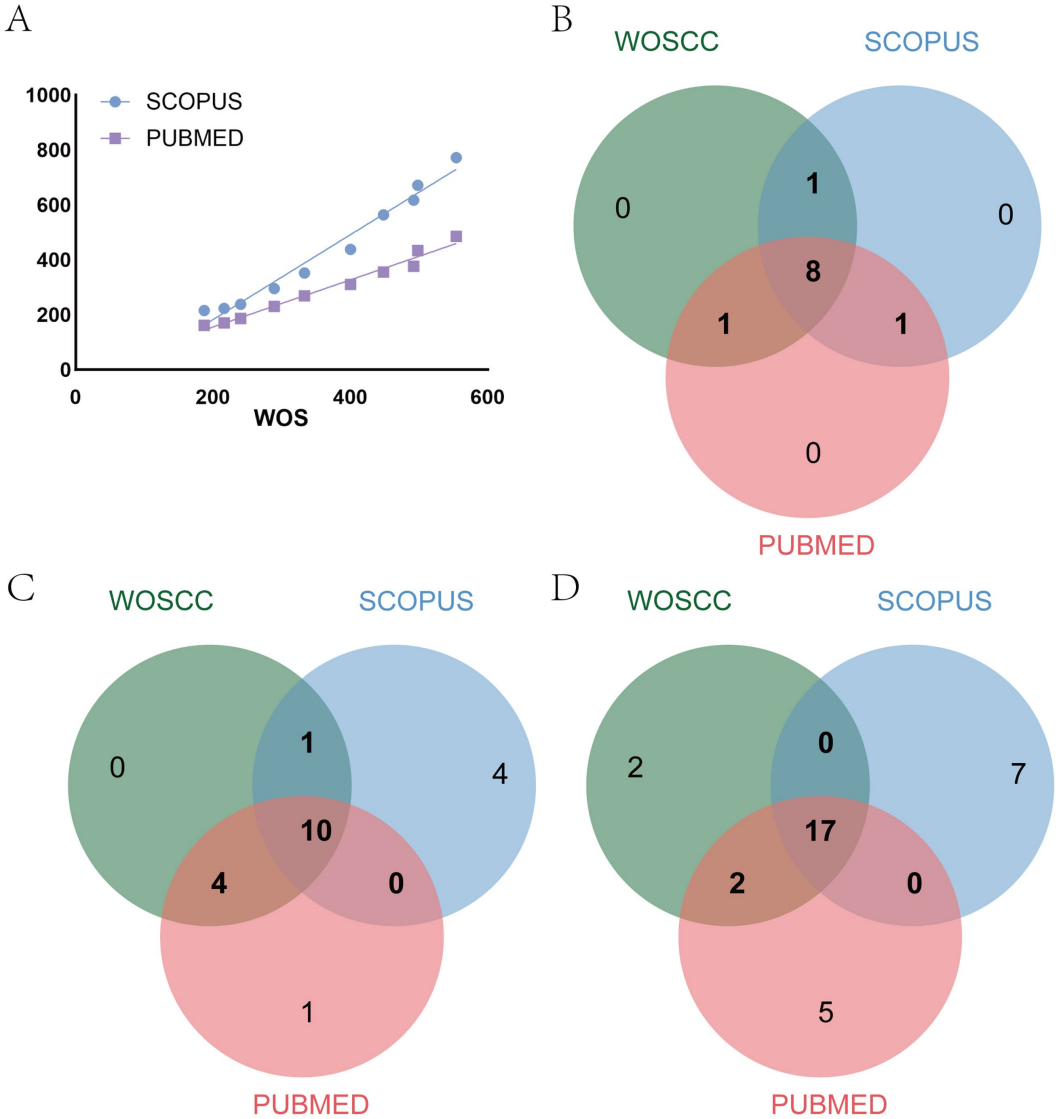
**(A)** Correlation analysis of annual publication trends in different databases; **(B)** Venn charts of the top 10 countries in terms of the number of published articles; **(C)** Venn diagrams of the top 15 keywords; **(D)** Venn diagrams of core journals.

Employing identical search strategies and inclusion/exclusion criteria, we retrieved 4,385 articles from Scopus and 2,976 articles from PubMed. The annual publication trends across the three databases were relatively consistent, with Pearson correlation coefficients of 0.9857 between WoSCC and Scopus, and 0.9876 between WoSCC and PubMed (Figure 9A). Both correlations were statistically significant (*p* < 0.001). The distribution of key countries across databases remained stable. The Jaccard similarity coefficients between WoSCC and Scopus, and between WoSCC and PubMed, were both 0.818, indicating substantial overlap (Figure 9B). The top 15 keywords (Figure 9C) also demonstrated high similarity (WoSCC vs. Scopus: 0.5789; WoSCC vs. PubMed: 0.875). Although the number of core journals exhibited differing trends across databases, their compositional distributions showed relatively consistent patterns (WoSCC vs. Scopus: 0.6071; WoSCC vs. PubMed: 0.7308; Figure 9D). Spearman’s correlation analysis revealed high correlations (*p* < 0.05) in the relative rankings of the top 15 keywords across databases, while relative country rankings exhibited some divergence (WoSCC vs. Scopus: *p* = 0.0003; WoSCC vs. PubMed: *p* = 0.0667). These findings indicate that despite variations in the datasets retrieved by each database, the macro-level trends, relative importance of countries/journals, and core themes remain relatively stable.

## 4 Discussion

### 4.1 General information

In recent years, astrocytes have emerged as a focal point in cognitive impairment research, evidenced by a marked increase in scholarly publications. The United States and China have emerged as leading contributors to this field, potentially driven by the high prevalence of cognitive impairment in both populations. Epidemiological data from 2019 confirm that these nations rank among those with the highest incidence rates of cognitive impairment (GBD 2019 Dementia Forecasting Collaborators, 2022). The establishment of multiple high-impact factor journals in this domain reflects significant scientific advancements. Although several Chinese institutions demonstrate notable publication productivity, their academic impact requires further development. Professor Henrik Zetterberg has been identified as the most prolific and highly cited researcher, with his team primarily investigating the astrocytic biomarker GFAP for clinical diagnosis and prognosis across various cognitive disorders (Graham et al., 2025; Pereira et al., 2021).

### 4.2 Hotspots and trends

**Figure 10:**
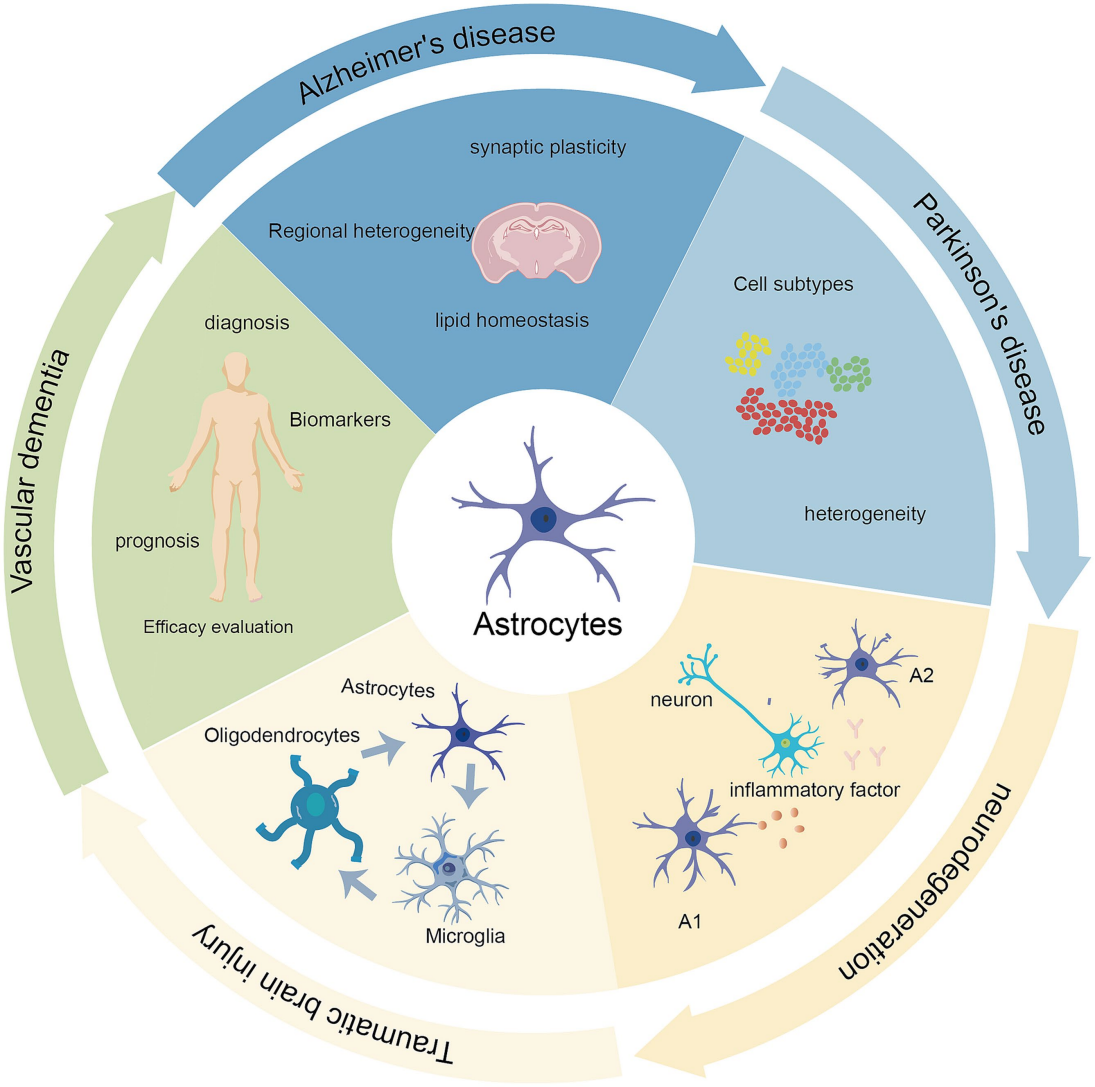
Graphical summary of research hotspots.

Through bibliometric analysis of keywords and co-cited literature, we have identified research hotspots and trends concerning astrocytes in cognitive impairment. The main areas of concentration are disease and mechanisms. Figure 10 illustrates the key research hotspots and trends in this field.

As shown in Figures 6–8, neuroinflammation has been identified as a long-standing research hotspot, and it is expected to remain a focal point of research in the future. Neuroinflammation functions as a critical neuroprotective mechanism during the initial phases of cerebral injury (Wyss-Coray and Mucke, 2002). However, persistent inflammatory stimuli can impede neural repair and ultimately lead to cognitive dysfunction (Kempuraj et al., 2016). Highly cited and burst literature by Liddelow et al. (2017) and Kwon and Koh (2020) have provided important insights into neuroinflammation mediated by reactive astrocytes. Liddelow SA’s research classifies reactive astrocytes into two types: A1 type, which is neurotoxic by promoting the secretion of pro-inflammatory cytokines, and A2 type, which exerts neuroprotective effects by upregulating neurotrophic factors. This suggests that inhibiting the A1 phenotype and promoting the A2 phenotype is an important strategy for treating cognitive impairment. Hyuk Sung Kwon’s research further summarizes specific activation pathways of protective astrocytes, including the SHP2/Ras/ERK, TGFβ, interferon (IFN)-*γ* signaling, and STAT3 pathways, providing guidance for developing targeted therapeutic approaches. Notably, AS are influenced by specific pathological environments, disease stages, and regional specificity in the brain. The binary classification of AS has limitations due to the inherent heterogeneity in their gene expression profiles and functional diversity, posing significant challenges for identifying and characterizing different subpopulations (Jiwaji and Hardingham, 2022). This necessitates a deeper exploration of AS heterogeneity. As shown in Figure 8C, single-cell sequencing has become an important technique for addressing this challenge.

Further analysis of the timeline indicates that earlier studies typically employed scRNA-seq to explore the diversity of AS types and target regulatory genes (Ceyzériat et al., 2018). In recent years, the introduction of pseudotime analysis and spatial transcriptomics has provided new perspectives for studying the heterogeneity of astrocytes in both temporal and spatial dimensions (Butler et al., 2018). The scRNA-seq has also advanced research into cellular communication, as evidenced by keyword clustering analysis (Figure 6A), which indicates that cellular communication has become a prominent research focus within this field. This signals a significant shift in research within the field—from the isolated deconstruction of astrocytes themselves toward understanding their function within the intact neurovascular unit (NVU) and indeed the broader brain ecosystem network. The recurrent presence of microglia and oligodendrocytes within this cluster underscores the pivotal role of glial cells in this process. Glial cells, together with neurons and microvessels, constitute the NVU. Their dynamic interactions maintain stable cerebral blood flow and blood–brain barrier function, representing key therapeutic targets for cognitive impairment (Kapasi and Schneider, 2016). The top-most-cited publication reveals that microglia can release pro-inflammatory cytokines to induce astrocyte differentiation toward a neurotoxic phenotype (Liddelow et al., 2017). Conversely, astrocytes modulate microglial function by secreting cytokines such as IL-3, thereby enhancing microglial phagocytic capacity and initiating immune responses (Lian et al., 2016). Oligodendrocytes are responsible for myelin generation, and myelin destruction can lead to cognitive impairments (Wang et al., 2023). Activated astrocytes exhibit dual regulatory effects on oligodendrocyte dynamics: while promoting oligodendrocyte apoptosis through TNF-*α* release, they simultaneously facilitate the differentiation, maturation, and inflammatory site recruitment of oligodendrocyte precursor cells (OPCs) for neural repair (Domingues et al., 2016; Madsen et al., 2016; Moyon et al., 2015). A comprehensive understanding of these cellular interactions is essential for elucidating the molecular mechanisms underlying cognitive disorders and developing targeted therapeutic interventions. The research of Linnerbauer et al. (2020) began to show an explosive trend in 2022. It summarized the interactions between astrocytes and microglia, oligodendrocytes, neurons, endothelial cells, etc., and became an important reference for studying this mechanism.

Our atlas shows that astrocytes are involved in the progression of various cognitive disorders, including AD, VD, PD, and traumatic brain injury (TBI). Among these, Alzheimer’s disease occupies the most prominent position, which is consistent with its epidemiological burden as it has become the predominant type of cognitive impairment globally (Xiong et al., 2023). The bibliometric atlas shows strong thematic connections among these diseases. This suggests that despite their diverse etiologies, they may converge on a final common pathway mediated by astrocyte dysfunction. Keyword and co-citation clustering analyses further elucidate several key pathological mechanisms, including neuroinflammation, oxidative stress, mitochondrial dysfunction, glutamate homeostasis, and immunoregulation. Among these, synaptic dysfunction stands out as a particularly prominent area, having become a focal point of recent research. These findings provide a theoretical basis for exploring broad-spectrum therapeutic strategies targeting AS. Additionally, the frequent occurrence of the hippocampus suggests that it, as a critical node for learning and memory processing, has become a key brain region of interest in AS-mediated cognitive impairments (Basu and Siegelbaum, 2015).

### 4.3 Challenges in clinical translation

Current research remains predominantly focused on basic studies, with limited clinical investigations concentrated on diagnosis and prediction. As demonstrated by Keyword Cluster 7, AS has progressively emerged as a biomarker for diagnosing and addressing cognitive impairment. Relevant clinical studies indicate that AS biomarkers GFAP and S100B in blood and cerebrospinal fluid can reflect the severity of cognitive dysfunction in AD and VD (Agnello et al., 2025; Ehtewish et al., 2023; Hosoki et al., 2023). However, given the complex mechanisms underlying cognitive impairment, no single biomarker can comprehensively reflect disease status. Future research should focus on integrating machine learning techniques to identify optimal biomarker combinations for diagnosis, prognostic assessment, and treatment efficacy monitoring. Furthermore, basic research aims to serve clinical practice. Although animal studies have demonstrated the modulatory effects of certain drugs on AS (Deng et al., 2024), clinically applicable targeted therapies remain lacking. Current research has shown that repairing or replacing damaged astrocytes could be a key strategy for treating cognitive impairments (Hastings et al., 2022; Verkhratsky et al., 2023), but this approach has not yet been clinically validated. Surprisingly, researchers have successfully implemented microglial cell transplantation therapy in human subjects to treat adult-onset leukoencephalopathy with axonal spheroids and pigmented glia, providing a paradigm for the clinical realization of astrocyte transplantation therapy (Wu et al., 2025).

## 5 Conclusion

This study conducted a visual analysis of literature within the WOSCC over the past decade concerning cognitive impairment and astrocytes. Our findings reveal the primary diseases under investigation within the field, confirming the hippocampus as a key brain region and neuroinflammation as a critical pathological factor. This provides significant insights and reference points for subsequent research. Future research may focus on two primary directions: firstly, leveraging omics technologies such as single-cell sequencing to investigate neuroinflammation and intercellular interactions in depth, thereby deepening our understanding of astrocytes in the pathogenesis of cognitive impairment and subsequently developing targeted therapeutic strategies; secondly, further exploring the clinical application value of AS in cognitive impairment, including prediction, diagnosis, prognosis assessment, and treatment.

## 6 Limitation

This study has some inherent limitations of bibliometric analysis. The first is database coverage bias. We conducted robustness tests through multiple academic databases to mitigate potential coverage bias. However, relying solely on WOSCC and restricting English publications for literature retrieval inevitably leads to the omission of relevant research, especially for research contributions from non-English-speaking countries. The second issue is citation bias. Papers from high-impact factor journals and well-known authors are more likely to be cited. The top 10 co-cited documents are almost all from journals with an IF greater than 10. Finally, due to the time required for accumulation and indexing, newer publications may have fewer citations, which may underestimate emerging trends and cause lag effects.

## Data Availability

The original contributions presented in the study are included in the article/Supplementary material, further inquiries can be directed to the corresponding authors.
