# Supplementary material for: Research trends and frontiers of astrocytes in cognitive impairment: a bibliometric analysis from 2015 to 2024
Source: Front Aging Neurosci. 2026 Jan 13;17:1708008. doi: 10.3389/fnagi.2025.1708008 (PMC12835277; doi:10.3389/fnagi.2025.1708008)
Supplement: Supplementary file 1 [file Table_1.DOC]

PUBMED

("Astrocytes"[Mesh] OR

(Astrocytes[tiab] OR Astrocyte[tiab] OR "Astroglia Cells"[tiab] OR "Astroglia Cell"[tiab] OR "Cell, Astroglia"[tiab] OR "Astroglial Cells"[tiab] OR "Astroglial Cell"[tiab] OR "Cell, Astroglial"[tiab] OR Astroglia[tiab] OR Astroglias[tiab] OR Astroglial[tiab]))

AND("Cognitive Dysfunction"[Mesh] OR( "Cognitive Dysfunction"[tiab] OR "Cognitive Dysfunctions"[tiab] OR "Dysfunction, Cognitive"[tiab] OR "Dysfunctions, Cognitive"[tiab] OR "Cognitive Disorder"[tiab] OR "Cognitive Disorders"[tiab] OR "Disorder, Cognitive"[tiab] OR "Disorders, Cognitive"[tiab] OR "Cognitive Impairments"[tiab] OR "Cognitive Impairment"[tiab] OR "Impairment, Cognitive"[tiab] OR "Impairments, Cognitive"[tiab] OR "Mild Cognitive Impairment"[tiab] OR "Cognitive Impairment, Mild"[tiab] OR "Cognitive Impairments, Mild"[tiab] OR "Impairment, Mild Cognitive"[tiab] OR "Impairments, Mild Cognitive"[tiab] OR "Mild Cognitive Impairments"[tiab] OR "Cognitive Decline"[tiab] OR "Cognitive Declines"[tiab] OR "Decline, Cognitive"[tiab] OR "Declines, Cognitive"[tiab] OR "cognitive deficit"[tiab] OR "cognitive deficits"[tiab] OR dementia[tiab] OR dementias[tiab]))

SCOPUS

( TITLE-ABS-KEY ( Astrocytes OR Astrocyte OR "Astroglia Cells" OR "Astroglia Cell" OR "Cell, Astroglia" OR "Astroglial Cells" OR "Astroglial Cell" OR "Cell, Astroglial" OR Astroglia OR Astroglias OR Astroglial ) )

AND(TITLE-ABS-KEY ( "Cognitive Dysfunction" OR "Cognitive Dysfunctions" OR "Dysfunction, Cognitive" OR "Dysfunctions, Cognitive" OR "Cognitive Disorder" OR "Cognitive Disorders" OR "Disorder, Cognitive" OR "Disorders, Cognitive" OR "Cognitive Impairments" OR "Cognitive Impairment" OR "Impairment, Cognitive" OR "Impairments, Cognitive" OR "Mild Cognitive Impairment" OR "Cognitive Impairment, Mild" OR "Cognitive Impairments, Mild" OR "Impairment, Mild Cognitive" OR "Impairments, Mild Cognitive" OR "Mild Cognitive Impairments" OR "Cognitive Decline" OR "Cognitive Declines" OR "Decline, Cognitive" OR "Declines, Cognitive" OR "cognitive deficit" OR "cognitive deficits" OR dementia OR dementias )

)
